# Cost-effectiveness of using protons for breast irradiation aiming at minimizing cardiotoxicity: A risk-stratification analysis

**DOI:** 10.3389/fmed.2022.938927

**Published:** 2022-08-25

**Authors:** Guo Li, Yun-Fei Xia, Yi-Xiang Huang, Deniz Okat, Bo Qiu, Jerome Doyen, Pierre-Yves Bondiau, Karen Benezery, Jin Gao, Chao-Nan Qian

**Affiliations:** ^1^Department of Radiation Oncology, Affiliated Cancer Hospital and Institute of Guangzhou Medical University, Guangzhou, China; ^2^Department of Radiation Oncology, Guangzhou Concord Cancer Center, Guangzhou, China; ^3^State Key Laboratory of Oncology in South China and Collaborative Innovation Center for Cancer Medicine, Sun Yat-sen University Cancer Center, Guangzhou, China; ^4^Department of Radiation Oncology, Sun Yat-sen University Cancer Center, Guangzhou, China; ^5^Department of Health Management, Public Health Institute of Sun Yat-sen University, Guangzhou, China; ^6^Department of Finance, Hong Kong University of Science and Technology, Kowloon, Hong Kong SAR, China; ^7^Department of Radiation Oncology, Antoine Lacassagne Cancer Center, University of Nice-Sophia, Nice, France; ^8^Mediterranean Institute of Proton Therapy, Antoine Lacassagne Cancer Center, University of Nice-Sophia, Nice, France; ^9^Division of Life Sciences and Medicine, Department of Radiation Oncology, The First Affiliated Hospital of University of Science and Technology of China, University of Science and Technology of China, Hefei, China

**Keywords:** proton beam therapy, breast cancer, cost-effectiveness analysis, ischemic heart disease, Markov model

## Abstract

**Background:**

Incidental exposure of the heart to ionizing irradiation is associated with an increased risk of ischemic heart disease and subsequent fatality in patients with breast cancer after radiotherapy. Proton beam therapy can limit the heart dose in breast irradiation to a negligible level. However, compared with conventional photon modality, proton breast irradiation is more expensive. In this study, we performed cost-effectiveness analyses to identify the type of patients who would be more suitable for protons.

**Methods:**

A Markov decision model was designed to evaluate the cost-effectiveness of protons vs. photons in reducing the risk of irradiation-related ischemic heart disease. A baseline evaluation was performed on a 50-year-old woman patient without the preexisting cardiac risk factor. Furthermore, risk-stratification analyses for photon mean heart dose and preexisting cardiac risk were conducted on 40-, 50-, and 60-year-old women patients under different proton cost and willingness-to-pay (WTP) settings.

**Results:**

Using the baseline settings, the incremental effectiveness (protons vs. photons) increased from 0.043 quality-adjusted life-year (QALY) to 0.964 QALY when preexisting cardiac risk increased to 10 times its baseline level. At a proton cost of 50,000 US dollars ($), protons could be cost-effective for ≤ 60-year-old patients with diabetes and ≤50-year-old patients with grade II–III hypertension at the WTP of China ($37,653/QALY); for ≤ 60-year-old patients with diabetes and ≤ 50-year-old patients with grade II–III hypertension or ≥ 2 major cardiac risk factors at a WTP of $50,000/QALY; and for ≤ 60-year-old patients with diabetes, grade II–III hypertension or ≥ 2 major cardiac risk factors and ≤ 50-year-old patients with total cholesterol ≥ 240 mg/dL at a WTP of $100,000/QALY.

**Conclusion:**

Patients' preexisting cardiac risk status was a key factor affecting the cardiac benefits gained from protons and should therefore be a major consideration for the clinical decision of using protons; cost-effective scenarios of protons exist in those patients with high risk of developing cardiac diseases.

## Introduction

Breast cancer (BC) has surpassed lung cancer as the most commonly diagnosed cancer. Every year, an estimated 2.3 million people are diagnosed with BC worldwide, accounting for 11.7% of all new cancer cases (1, 2). Radiotherapy is an integral part of the multidisciplinary management of BC and is routinely recommended as a standard therapeutic procedure for patients with BC after breast-conserving surgery and for patients with BC with an intermediate or high risk of locoregional recurrence after mastectomy (3, 4). However, the role of postoperative radiotherapy in improving overall survival is still debatable. It could be partly due to the incidental exposure of the heart to ionizing irradiation, which increases the risk of ischemic heart disease (IHD) and subsequent fatality (5, 6). In the era of photon irradiation, irradiation-related IHD is recognized as the leading cause of non-cancer death and a main deteriorating factor of life quality in BC survivors (7). New technologies with lower cardiotoxicity are urged to optimize the irradiation efficacy for patients with BC.

Proton beam therapy is a rapidly evolving particle irradiation modality with superior dose distribution owing to protons' “Bragg peaks” (8). Its advanced form, the intensity-modulated proton radiation therapy, can limit the mean heart dose (MHD) to a level of < 0.5 Gray (Gy) while maintaining excellent tumor control in breast irradiation, whereas conventional photon modality often leads to an MHD range of 5–16 Gy (9–11). However, proton irradiation is also associated with an increase in medical costs, with a current cost ratio (cost of protons/cost of photons) ranging from 3.2 to 4.8. The uncertainty in cost-effectiveness makes the use of proton irradiation a controversial issue, especially for patients with BC in developing countries. Cost-effectiveness analysis (CEA) is urgently needed to select appropriate patients for protons and save medical resources (12).

Previous studies indicated that protons could be cost-effective for patients with BC if they could reduce photon-induced IHD risk to a certain extent; depending mainly on the estimated heart dose in photon irradiation and the patient's preexisting cardiac risk status (13, 14). A population-based case-control study by Darby et al. found a linear relationship between MHD and the incidence of IHD and reported that each Gy increase in MHD was correlated with a 7.4% increase in IHD risk, for which the preexisting cardiac risk acted as the base (15). Preexisting cardiac risk in patients with BC may be due to “traditional” cardiac risk factors (CRFs; such as abnormal lipids, high blood pressure, diabetes, and smoking) or the potential use of anticancer agents known to affect the heart (such as anthracycline, trastuzumab, and taxanes) (16–18). Therefore, to identify which groups of patients with BC could cost-effectively benefit from protons, both the photon heart dose and the patient's preexisting cardiac risks should be stratified. However, no such risk-stratification study has been conducted.

Herein, we designed a CEA model to evaluate the cost-effectiveness of protons vs. photons in terms of reducing the irradiation-related IHD risk, in light of photon MHD and preexisting cardiac risk. Our aim was to estimate the “cost-effective” risk groups to facilitate the medico-economic decision-making on protons.

## Methods

### Model design

The TreeAge Pro 2018 software (TreeAge, Williamstown, Massachusetts) was used for model building and statistical analyses. Two-arm Markov decision model was built based on the following assumptions: (1) the advantage of protons over photons lies in its ability to reduce the lifetime risk of IHD (15), (2) a constant 0.5 Gy of MHD was assumed for all proton irradiations, in comparison to different MHD yielded in photon irradiations (9, 13), (3) tumor control and other treatment toxicity profiles were identical between the two strategies (8–11), (4) the lifetime IHD risk was identical between the non-irradiated BC population and the general population.

**Figure 1:**
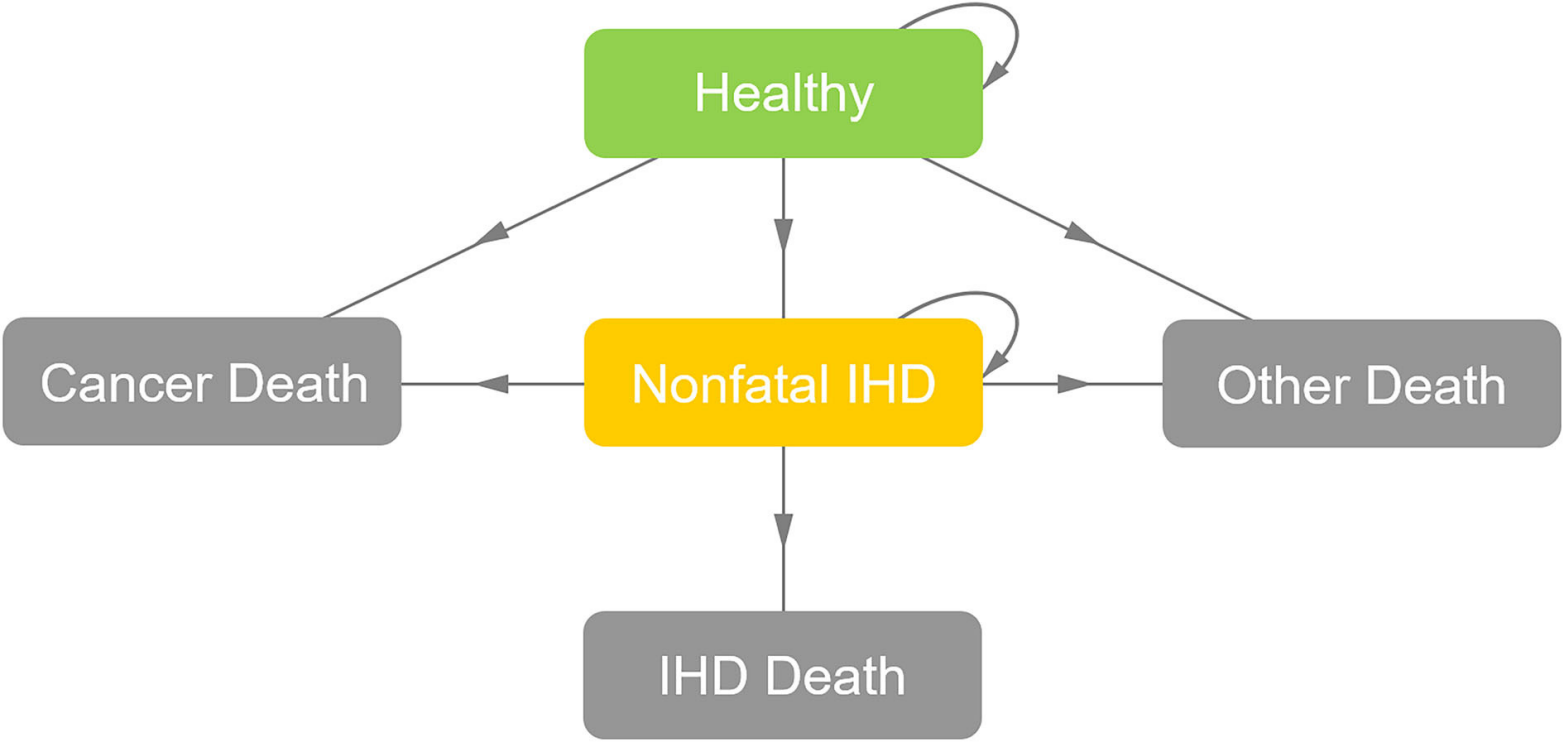
General structure of the Markov model. After radiotherapy, the patient might be in a state of “healthy,” “nonfatal IHD,” or “death” (“IHD death,” “cancer death,” or “other death”). For each cycle, if the patient was in the state of “healthy,” she might stay in the state of “healthy,” develop into the state of “non-fatal IHD” or develop into the “death” states; if the patient was in the state of “non-fatal IHD,” she might stay in the state of “non-fatal IHD” or develop into the “death” states; and if the patient was in the absorbing “death” states, the loop operation would be terminated. IHD, ischemic heart disease.

The general structure of the Markov model is illustrated in Figure 1. The following five states, namely, “healthy,” “non-fatal IHD,” “IHD death,” “cancer death” and “other death,” were used to simulate the natural process of patients with BC after radiotherapy. The two strategies experienced the same “cancer death” risk in the first 5 years after radiotherapy, leading to a background 5-year “cancer death” rate of 6% (19). The risk of “other death” was calculated based on the 2016 Life Tables of the United States (20). A 1-year cycle length was used and the Markov models were cycled from the end of radiotherapy until the age of 80, to evaluate the cost-effectiveness over a lifetime horizon.

### Model set-ups for ischemic heart disease risk

The risk of IHD includes the risk of fatal IHD (“IHD death”) and the risk of “nonfatal IHD” (including non-fatal acute myocardial infarction, angina pectoris, and ischemic heart failure) (21, 22). CEA models were set separately for 40-, 50- and 60-year-old patients with BC to simulate their different IHD occurrences. The model set-ups for IHD risk followed three steps: First, the occurrence of “IHD death” in the model was set according to the annual death risk data in the report of Darby et al. (15) (Supplementary Table 1). Second, the lifetime risk of “non-fatal IHD” was set to 5 times, 4 times, 2 times, and 1 time the lifetime risk of “IHD death” for the age levels of <50, 50–59, 60–69, and <70, respectively (15). Third, the final cumulative IHD death risk and total IHD risk (to age 80) were calibrated to be exactly the same as the cumulative risk data reported by Darby et al. (15) (Supplementary Tables 2, 3) overall, irradiation-related IHD risk increased linearly with the MHD by 7.4% per Gy.

### Baseline set-ups

Baseline evaluation was performed on a 50-year-old postmenopausal woman patient who was diagnosed with stage II (T2N1M0) left-sided invasive ductal carcinoma and had no preexisting CRF. The patient received breast-conserving surgery and was awaiting postoperative breast irradiation to reduce the risk of tumor recurrence. We initially assumed that breast irradiation using photons led to an MHD of 5 Gy, compared to 0.5 Gy when using protons. Accordingly, the IHD death risk and total IHD risk to the age of 80 were set to 2.0 and 4.6% for proton strategy, and 2.7 and 6.1% for photon strategy (15).

### Risk-stratification and major cardiac risk factor strata

Risk-stratification analyses were conducted based on the photon MHD and the preexisting cardiac risk. Using the baseline case as a contrast (a photon MHD of 5Gy, without preexisting CRF), the photon MHD ranging from a minimum of 1 Gy to a maximum of 16 Gy was analyzed; and the preexisting cardiac risk ranging from the baseline cardiac risk level to its 10 times was analyzed. To identify the “cost-effective” scenarios, the minimum photon MHDs (the thresholds beyond which protons would be considered “cost-effective”) were estimated for patients with BC at different preexisting cardiac risk levels (a range from the lifetime IHD risk in non-irradiated women without CRF to its 12 times).

The general-population IHD risk data from the Framingham Heart Study were used to estimate the preexisting lifetime IHD risk for patients with BC in selected CRF strata (23, 24). Lifetime IHD risk stratified by the major CRFs, including total cholesterol ≥240 mg/dL, grade II–III hypertension (systolic blood pressure ≥160 mm Hg or diastolic blood pressure ≥100 mm Hg), smoker, and diabetes, are summarized in Supplementary Table 4. Patients with BC in the selected major CRF strata were included in the “cost-effective” groups if the required minimum photon MHD ≤ 5 Gy.

### Costs and utilities

All costs were adjusted to US dollars ($), using a Sino-US exchange rate of $1 = 6.45 RMB (average value in the year 2021). The treatment regimens of the two compared strategies were similar except for the irradiation modality. Our protocol used for radiotherapy cost estimation was as previously reported (25–27). The prescription dose for postoperative breast irradiation was 50 Gy (at 2 Gy per fraction) to the breast/chest wall and lymph node regions plus an additional 16 Gy (at 2 Gy per fraction) to the tumor bed/mastectomy incision (28). The cost of protons was assumed to be $50,000 for breast irradiation using intensity-modulated proton radiation therapy. Additional $40,000, $30,000, and $20,000 were adopted to simulate proton cost reduction. A constant $12,000 was used as the cost for photon breast irradiation using intensity-modulated photon-radiation therapy. We assumed that patients with “non-fatal IHD” would undergo a one-time percutaneous coronary intervention to treat ischemic symptoms, and the cost for percutaneous coronary intervention was estimated as $10,000, simulating the costs for interventional cardiology materials (1.5 stents, catheters, guide wires, balloons, and microcatheters) and other costs for operation, an anticoagulant drug, blood tests, and hospitalization (29). The annual treatment costs for non-fatal IHD were estimated as $2,000, simulating medical care for ischemic symptoms or the maintenance after percutaneous coronary intervention (including a daily b-blocker, aspirin, angiotensin-converting enzyme inhibitor, and statin, in addition to tests at diagnosis of both rest and stress electrocardiogram, hemoglobin level, and fasting lipid panel) (30). The follow-up cost per year was assumed as $1,000, simulating a set of examinations, including hematologic and biochemistry profiles, magnetic resonance imaging of breast, chest radiography, and abdominal ultrasonography.

The utility was adjusted to quality-adjusted life-year (QALY) using a health state utility value (HSUV). The HSUV for the states of “healthy” was standardized as 0.95, simulating a disease-free state after radical anticancer treatment (31), and the HSUV for the states of “non-fatal IHD” was standardized as 0.695, simulating the cardiovascular symptoms caused by non-fatal IHD (angina pectoris) (32). Half-cycle corrections were performed to minimize discretization errors in the continuous Markov process (33). Costs and QALY were discounted at an annual rate of 3% to statistically isolate time preference effects (34).

### Analyses

Markov cohort analysis was performed to examine the state probabilities of the two compared strategies in the modeling. Probabilistic sensitivity analysis was performed to examine the robustness of the model in light of a joint uncertainty for cost and utility parameters by running over 50,000 iteration trials. The 90% confidence interval of the parameters was identified. The parameters of utility were tested using beta distributions, and the parameters of cost were assessed using uniform distributions. A tornado diagram was used to evaluate the influences of the parameters on cost-effectiveness by varying each parameter over the 90% confidence interval identified in probabilistic sensitivity analyses.

The outcome measure of the CEA model was the incremental cost-effectiveness ratio (ICER), which represents the ratio of the difference in costs to the difference in treatment effectiveness (incremental cost/incremental effectiveness) between proton strategy and photon strategy. A Chinese societal perspective was adopted. According to the World Health Organization guidelines, a strategy is defined as cost-effective if the ICER value is below three times the gross domestic product (GDP) per capita (35). Thus, $37,653/QALY (three times the Chinese GDP per capita in 2021) was applied as the societal willingness-to-pay (WTP) threshold of China to estimate the cost-effective scenarios of protons in this study (36). The other two published acceptable WTP thresholds ($50,000/QALY and $100,000/QALY) were also adopted (37).

## Results

### Model robustness verification

**Figure 2:**
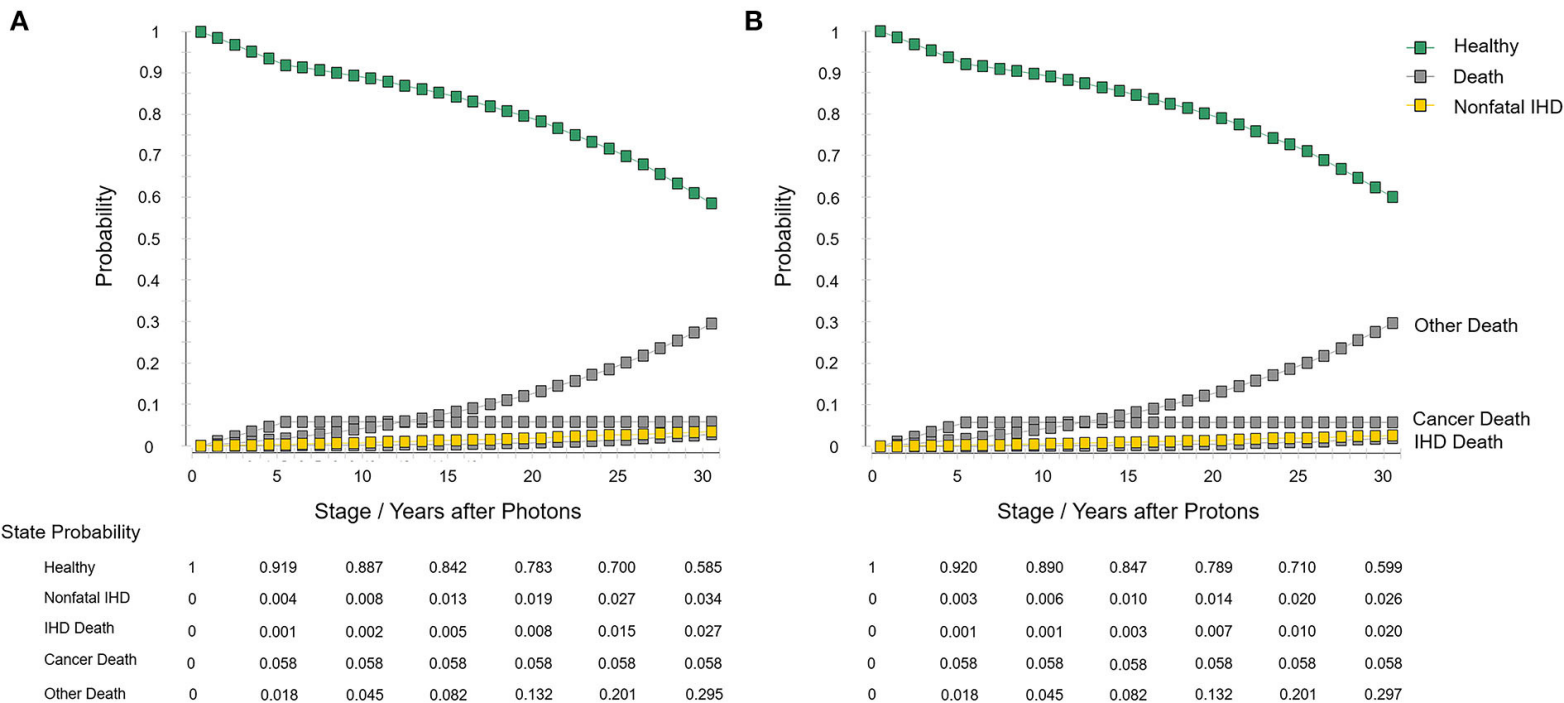
Markov cohort analyses with the baseline set-ups. **(A)** Photon strategy and **(B)** proton strategy. IHD, ischemic heart disease.

**Figure 3:**
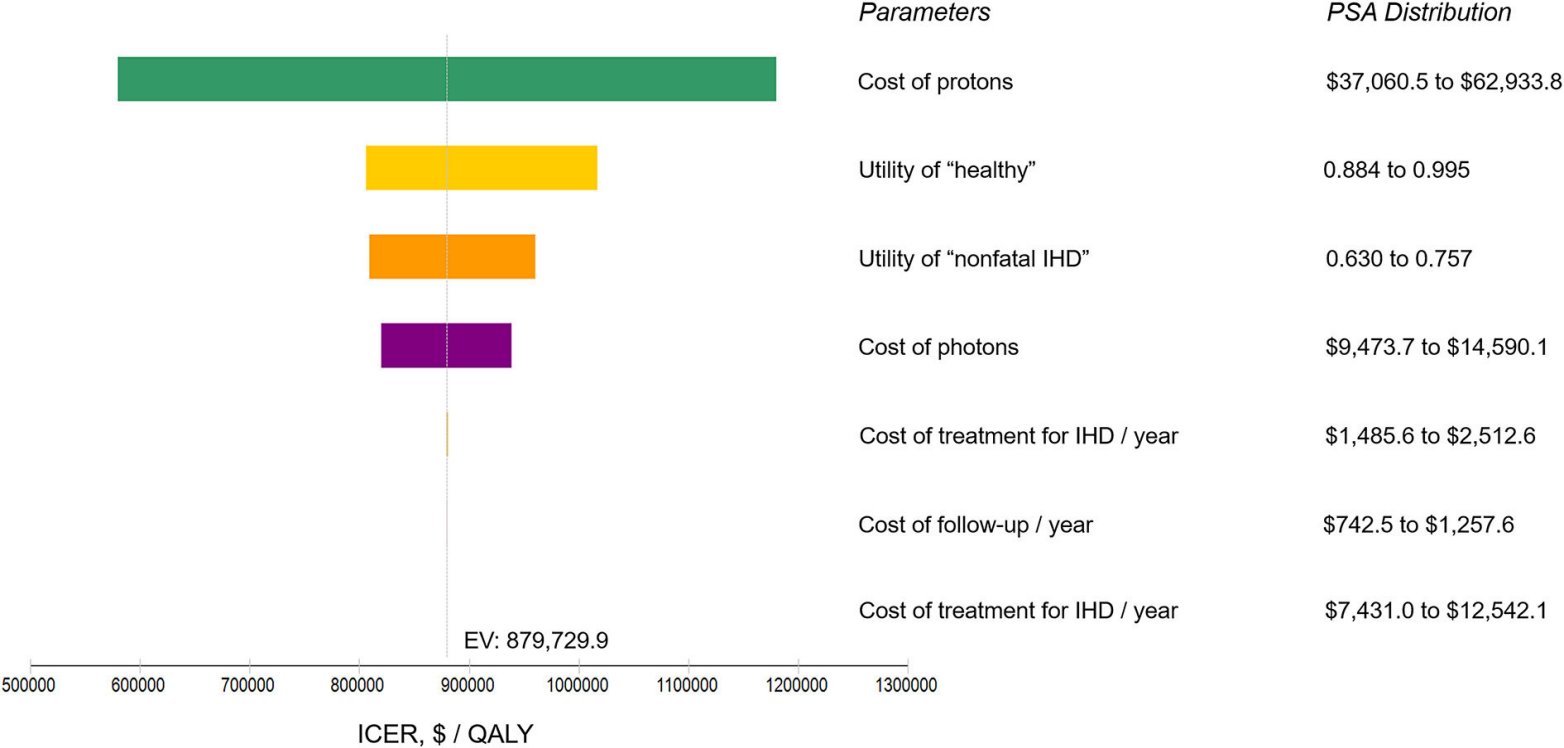
Tornado diagram analysis using the baseline set-ups. Illustrating the range of incremental cost-effectiveness ratio (ICER) when varying each parameter individually over a range of their 90% confidence interval identified in probabilistic sensitivity analysis (PSA). Influential parameters are listed in descending order according to their abilities in affecting the ICER. IHD, ischemic heart disease; $, US dollars; QALY, quality-adjusted life-year; EV, expected value.

The model information and set-ups are summarized in Table 1. The model robustness verification was conducted using the baseline set-ups. Figure 2 displays the results of Markov cohort analyses for the baseline case and confirmed that the 5-year “cancer death” risk was 5.8% for both the two strategies, and the cumulative IHD death risk and total IHD risk for 80-year-old was 2.0 and 4.6% for proton strategy, and 2.7 and 6.1% for photon strategy; corresponding to the data of Darby et al. (15). The tornado diagram examining the robustness of utility and cost parameters demonstrated that only the cost of protons had an obvious impact on ICER. When the cost of protons varied from $37,060.5 to $62,933.8, ICER ranged from $579,499.2/QALY to $1,179,828.2/QALY. The impact of the utility of “healthy,” the utility of “non-fatal IHD,” the cost of photons was relatively limited, and changing individual parameters did not lead to a notable change in ICER (Figure 3). For the baseline case, protons provided a 0.043 QALY at an additional cost of $37,915.1 and the ICER (protons vs. photons) was $879,729.9/QALY.

**Table 1:** Model information and set-ups.

**Parameters**	**Information**	**Source/reference**
**Evaluated treatment strategies**	Protons vs. photons	
**IHD risk**		
Annual risk of death from IHD	(Supplementary Table 1)	(15)
Cumulative risk of IHD death	(Supplementary Table 2)	(15)
Cumulative risk of IHD	(Supplementary Table 3)	(15)
Non-irradiated general-population IHD risk	(Supplementary Table 4)	(23, 24)
**Utilities, QALY**		
Healthy	0.950	(31)
Non-fatal IHD	0.695	(32)
Death	0	
**Cost** ^a^**, $**		
Protons	20,000 / 30,000 / 40,000 / 50,000	(25–27)
Photons	12,000	(25–27)
PCI	10,000	(29)
Treatment for non-fatal IHD / year	2,000	(30)
Follow-up / year	1,000	
**Baseline set-ups**		
Patient age	50-year-old	Assumption
Tumor stage	T2N1M0, Stage II	Assumption
Proton MHD	0.5 Gy	Assumption
Photon MHD	5 Gy	Assumption
Preexisting cardiac risk factor	None	Assumption
Proton cost, $	50,000	(25–27)
**Model basic set-ups**		
Cancer death risk	0.06 (5-year)	(19)
Other death risk	Life Tables 2016	(20)
Cycle length	1-year	
No. of cycles^b^	80–patient age	
Discount rate / year, %	3	(34)
Willingness-to-pay, $/QALY	37,653^c^ / 50,000 / 100,000	(36, 37)

MHD, mean heart dose; Gy, Gray; IHD, ischemic heart disease; QALY, quality-adjusted life-year; $, US dollars; PCI, percutaneous coronary intervention.

a All costs were derived from an institutional chart review.

b Markov models were to be cycled “80—patient age” times to evaluate the outcomes over a time-period from the end of radiotherapy to the age of 80. The number of cycles for 40-, 50-, and 60-year-old patients were 40, 30, and 20, respectively.

c $37,653/QALY is the societal willingness-to-pay threshold of China.

### Cost-effectiveness and photon mean heart dose/preexisting cardiac risk

**Figure 4:**
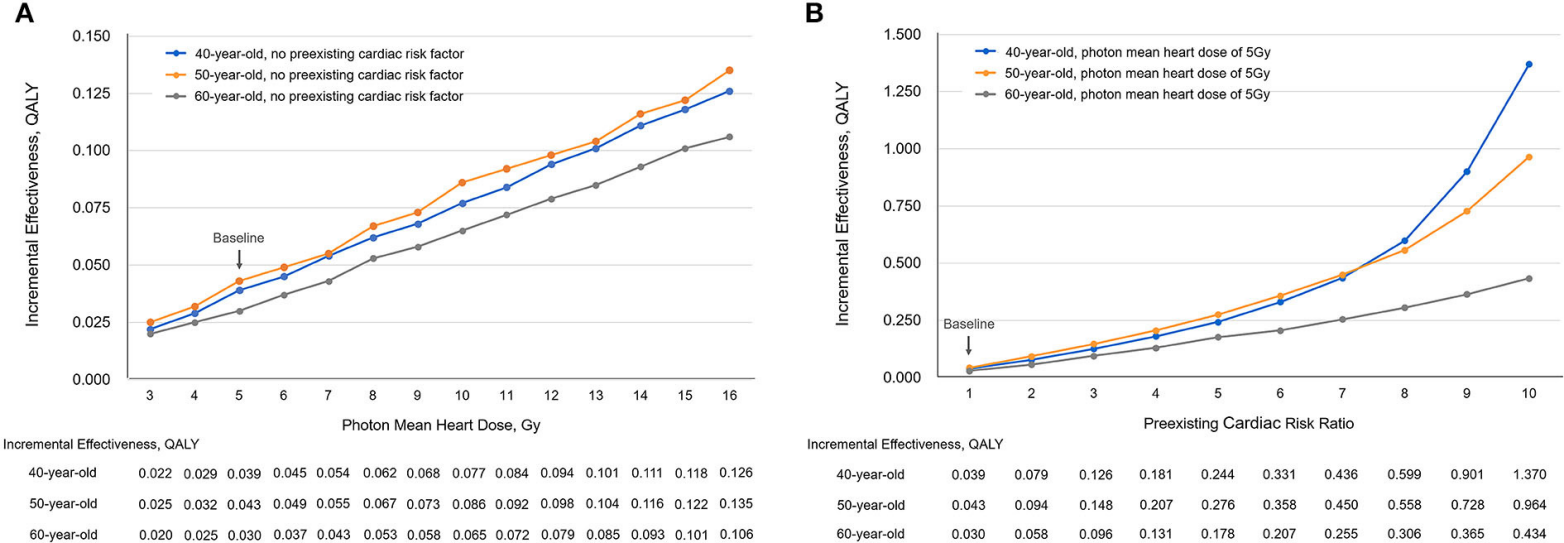
The incremental effectiveness (IE) changed with **(A)** photon mean heart dose and **(B)** preexisting cardiac risk. QALY, quality-adjusted life-year; Gy, Gray.

Using the baseline settings, the incremental effectiveness (protons vs. photons) ranged from 0.025 QALY to 0.135 QALY when the photon MHD varied from 3 to 16 Gy (Figure 4A), and the corresponding ICERs ranged from $1,528,810.0/QALY to $280,919.6 /QALY at a proton cost of $50,000; the incremental effectiveness ranged from 0.043 QALY to 0.964 QALY when the preexisting cardiac risk varied from baseline to 10 times baseline (Figure 4B), the corresponding ICERs ranged from $879,729.9/QALY to $39,299.2/QALY at a proton cost of $50,000. The ICERs at different proton cost levels ($50,000, $40,000, $30,000, and $20,000) were evaluated for 40-, 50- and 60-year-old patients and are listed in Supplementary Tables 5–10.

### Cost-effective scenarios

**Figure 5:**
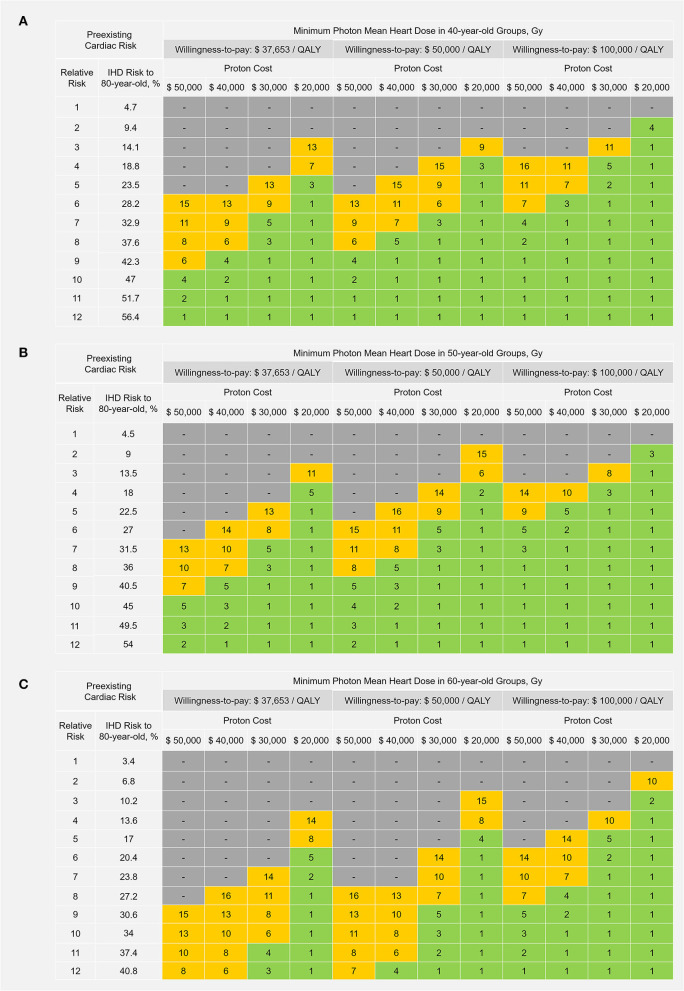
Cost-effective thresholds of photon mean heart dose in **(A)** 40-year-old. **(B)** 50-year-old; and **(C)** 60-year-old patients. Protons could be considered cost-effective to a patient if the estimated photon mean heart dose is ≥ the corresponding threshold value shown in the table. Green regions represent the photon mean heart dose range of 1–5 Gray (Gy); yellow regions represent the photon mean heart dose range of 6–16 Gy; and gray regions represent “not cost-effective” (the required minimum photon mean heart dose >16 Gy). $, US dollars; QALY, quality-adjusted life-year; IHD, ischemic heart disease.

Using different set-ups for WTP thresholds ($37,653/QALY, $50,000/QALY, and $100,000/QALY) and proton cost ($50,000, $40,000, $30,000, and $20,000), the “cost-effective” thresholds of photon MHD were estimated for patients at different preexisting cardiac risk levels and are displayed in Figures 5A–C for 40-, 50-, and 60-year-old patients. Protons were not cost-effective for patients without preexisting CRF under the costs and WTP settings of this study. Furthermore, 40-, 50- and 60-year-old patients in the selected major CRF strata were included in the “cost-effective” groups if the required minimum photon MHD ≤5 Gy (green regions in Figures 5A–C). Therefore, at the current proton cost ($50,000), protons could be cost-effective for ≤60-year-old patients with diabetes and ≤50-year-old patients with grade II–III hypertension at the WTP of China ($37,653/QALY); for ≤ 60-year-old patients with diabetes and ≤ 50-year-old patients with grade II–III hypertension or ≥2 major CRFs at a WTP of $50,000/QALY; and for ≤ 60-year-old patients with diabetes, grade II–III hypertension or ≥2 major CRFs and ≤ 50-year-old patients with total cholesterol ≥240 mg/dL at a WTP of $100,000/QALY.

## Discussion

BC is the most commonly diagnosed cancer worldwide and it is also a potential target for proton beam therapy (8–11, 38). This is the first CEA study stratifying both heart dose-related and preexisting cardiac risks to assess the cost-effectiveness of protons vs. photons in patients with BC. The substantial impact of the preexisting cardiac risk status on the cost-effectiveness was illustrated for the first time and further identified suitable patient groups that would benefit from protons, easing decision-making regarding the cost-effectiveness.

To examine the robustness of the model, Markov cohort analysis was applied as a calibration tool. It confirmed that the cancer-related survival rates for proton and photon strategy were identical, and the model-predicted IHD death risk and total IHD risk corresponded to the natural disease process, as previously reported (15). The tornado diagram evaluating the model parameter uncertainty showed that only the proton cost had an obvious impact on the ICER value. As such, the benefits of protons over photons in reducing IHD risk and the cost difference between the two irradiation modalities would be the determinants of the cost-effectiveness (ICER) in the CEA modeling; suggesting that the CEA model was robust. Besides, photon cost had only a minor impact on ICER. Upon comparing protons with three-dimensional conformal photon radiotherapy (a photon cost of $8,000), the ICER of the baseline case slightly increased from $879,729.9/QALY to $972,540.4/ QALY, suggesting that the conclusions would be similar when comparing protons to three-dimensional conformal photons.

A similar CEA study conducted by Mailhot Vega et al. also evaluated the IHD risk difference between protons and photons by using the data of Darby et al., but the effect of preexisting cardiac risk was only evaluated as the presence/absence of CRF in that study (13). As shown in the Framingham Heart Study, the lifetime IHD risk varied markedly with the presence of CRFs. For instance, among 50-year-old women, the lifetime IHD risk (to age 80) was 8.2% in those with all optimal risk factors, 20.5% in those at the overall level, 40.2% in those who had ≥ 2 major CRFs, and >57.3% in those with the presence of diabetes (24). Similarly, the preexisting CRFs in patients with BC could directly impact the cardiac benefits gained from protons (the absolute IHD risk reduction). However, the previous studies on proton decision-making have only emphasized dosimetric factors (proton/photon MHD), for example, by using NTCP model-based approach, while the patient's own cardiac risk has not been fully discussed or quantified (8, 39).

In our CEA modeling, both photon MHD and preexisting cardiac risk were quantified to estimate the absolute IHD risk reduction from protons. Using the baseline case as a contrast, our analyses showed that either an increase in photon MHD or an increase in preexisting cardiac risk could increase the incremental effectiveness and decrease the ICER, and preexisting cardiac risk has a greater impact on ICER than photon MHD (Figure 4). In current BC radiotherapy practice, the increasing use of prone positioning and breath-holding techniques have made high photon MHD less common (40), thus, preexisting cardiac risk status should be a major consideration for the clinical decision of using protons.

Under the costs and WTP settings of this study, we observed that only for the patients with major CRF burden, the cardiac benefits gained from protons could reach the “cost-effective” thresholds. Of note, diabetes was associated with the highest lifetime IHD risk among all single major CRF (Supplementary Table 4), thereby, was identified as a “cost-effective” risk factor to undergo protons (24, 41). From a perspective of Chinese society, a developing country with a GDP per capita close to the global average, protons could be cost-effective to ≤ 60-year-old patients with diabetes and ≤ 50-year-old patients with grade II–III hypertension. With appropriate settings, the current CEA model allows estimation of the cost-effective scenarios of protons for other BC populations. We observed that the cost-effective scenarios of protons expanded at the WTP thresholds of $50,000/QALY or $100,000/QALY (commonly adopted by the US society or other developed Western countries) (37). For instance, at a WTP of $100,000/QALY, protons could become cost-effective to≤ 60-year-old patients with diabetes, grade II–III hypertension, or ≥ 2 major CRFs and ≤ 50-year-old patients with total cholesterol ≥ 240 mg/dL. This indicated that a considerable proportion of patients with BC in Western countries may cost-effectively benefit from protons, although relevant specialized modeling is still required. Moreover, we also observed that protons became more cost-effective when we gradually decreased the proton cost from $50,000 to $20,000. Considering the opening of more proton centers, the introduction of a hypofractionated schedule, and proton technology upgrades, these potential future trends may drive a gradual reduction in proton cost and further increase the cost-effective scenarios (42, 43).

## Limitations

There were several limitations in this study worth addressing. First, the “cost-effective” risk groups of this study were estimated based on a series of assumptions. As to make a decision on whether to use protons for a specific BC patient, it still needs to perform CEA modeling based on personal dosimetric information, especially for those patients requiring special breast irradiation, which could result in a high level of MHD (such as internal mammary chain irradiation or synchronous bilateral BC irradiation) (18, 44). Second, data uncertainties did exist. It is worth mentioning that the incremental effectiveness in 40-year-old patients was not greater than those of 50-year-old patients in our CEA modeling, especially for those at low levels of preexisting cardiac risk (Figure 2). This phenomenon had also been observed in the study of Mailhot Vega et al. (13) and should be attributed to the survey of Darby et al., which excluded the patients with only angina when accounting for the total IHD risk. And, the potential inconsistency in IHD risk between the non-irradiated BC population and the general population and the IHD risk variation by race and region may affect our estimation for cost-effective scenarios to some extent, but this would not affect the model results based on specific IHD risk values. Third, due to the current data limitation, our analyses only involved 40-, 50- and 60-year-old women patients, and the modeling stopped at the age of 80, an age level close to the estimated average life expectancy of China (45). In the modeling, we found that the patient's preexisting cardiac risk status was the pivotal factor affecting the cost-effectiveness, and this cumulative cardiac risk is related to patient age and life expectancy. Theoretically, younger (<40-year-old) patients with BC could benefit more from protons compared to ≥ 40-year-old patients because they usually have higher cumulative lifetime IHD risk and more Markov cycles in the CEA modeling. And, the cost-effective scenarios of proton may exist in older (> 60-year-old) patients with BC, when older patients are at a high cardiac risk level and have a longer life expectancy. In addition, protons can be considered more cost-effective for male patients with BC than female patients with BC due to their higher level of preexisting IHD risk (23, 24). Fourth, as our study suggests, BC patients' preexisting cardiac risk status should be individually assessed before proton decision-making. However, the current cardiac risk prediction algorithms, such as the Framingham Risk Score (23), may hardly accurately estimate the cardiac risk for patients with BC who receive cardiotoxic anticancer agents (especially those treated with anthracycline-based chemotherapy or human epidermal growth factor receptor 2-targeted agents) (17, 18, 46, 47). Therefore, we call for the establishment of a preexisting cardiac risk prediction algorithm dedicated to patients with BC, preferably with variation by race and region, in future studies. Lastly, the irradiation-related IHD risk of this study was estimated using MHD based on the data of Darby et al. (15); however, MHD cannot reflect the doses to certain important cardiac substructures, such as the left descending artery and the left ventricle. When the MHD is low, the doses to these structures may be more significantly related to the cardiac risk, and protons, especially the intensity-modulated proton radiation therapy, could be more beneficial in decreasing doses to these structures compared to photons (48–50). Thus, future studies are also awaited to establish reliable dose-dependent models for a more concrete and individualized assessment of the irradiation-related cardiac risk.

## Conclusion

Using assumption-based CEA modeling and population-based cardiac risk data, the cost-effective scenarios of protons in patients with BC were estimated through the risk-stratification analyses for photon MHD and preexisting cardiac risk. The most striking findings of this study are that BC patient's preexisting cardiac risk status was a key factor affecting the cardiac benefits gained from protons and should therefore be a major consideration for the clinical decision of using protons; cost-effective scenarios of protons did exist in those patients with high risk of developing a cardiac disease.

## Data availability statement

The original contributions presented in the study are included in the article/Supplementary material, further inquiries can be directed to the corresponding author.

## Author contributions

C-NQ and GL had full access to all the data in the study and take responsibility for the integrity of the data and the accuracy of the data analysis, conception/design, and drafting of the manuscript. GL, Y-XH, P-YB, and JD: methodology. JG, Y-XH, DO, BQ, and KB: acquisition, analysis, or interpretation of data. Y-FX, P-YB, KB, and JD: critical revision of the manuscript for important intellectual content. C-NQ, Y-FX, and GL: obtained funding. C-NQ: supervision. All authors had read and approved the final version of the manuscript.

## Funding

This work was supported by grants to C-NQ from the National Natural Science Foundation of China (Nos. 82073220, 81872384, and 81672872); by grants to Y-FX from the National Nature Science Foundation of China (No. 81872464); and by grants to GL from the Guangzhou Major Clinical Technology Project of Affiliated Cancer Hospital & Institute of Guangzhou Medical University (No. 2060404).

## Conflict of interest

The authors declare that the research was conducted in the absence of any commercial or financial relationships that could be construed as a potential conflict of interest.

## Publisher's note

All claims expressed in this article are solely those of the authors and do not necessarily represent those of their affiliated organizations, or those of the publisher, the editors and the reviewers. Any product that may be evaluated in this article, or claim that may be made by its manufacturer, is not guaranteed or endorsed by the publisher.
