# Supplementary material for: Cost-effectiveness of using protons for breast irradiation aiming at minimizing cardiotoxicity: A risk-stratification analysis
Source: Front Med (Lausanne). 2022 Aug 25;9:938927. doi: 10.3389/fmed.2022.938927 (PMC9452743; doi:10.3389/fmed.2022.938927)
Supplement: Supplementary file 1 [file Data_Sheet_1.PDF]

Supplementary Material Contents

- **Supplementary Table 1** Data from population-based survey: annual risk of death from ischemic heart disease
- **Supplementary Table 2** Data from population-based survey: mean heart dose and cumulative risk of death from ischemic heart disease
- **Supplementary Table 3** Data from population-based survey: mean heart dose and cumulative risk of ischemic heart disease
- **Supplementary Table 4** Lifetime risk of ischemic heart disease for women in the Framingham Heart Study, by major cardiac risk factors
- **Supplementary Table 5** Cost-effectiveness and photon mean heart dose in 40-year-old patients without preexisting cardiac risk factor
- **Supplementary Table 6** Cost-effectiveness and preexisting cardiac risk in 40-year-old patients with a photon mean heart dose of 5 Gy
- **Supplementary Table 7** Cost-effectiveness and photon mean heart dose in 50-year-old patients without preexisting cardiac risk factor
- **Supplementary Table 8** Cost-effectiveness and preexisting cardiac risk in 50-year-old patients with a photon mean heart dose of 5 Gy
- **Supplementary Table 9** Cost-effectiveness and photon mean heart dose in 60-year-old patients without preexisting cardiac risk factor
- **Supplementary Table 10** Cost-effectiveness and preexisting cardiac risk in 60-year-old patients with a photon mean heart dose of 5 Gy

Supplementary Table 1 Data from population-based survey: annual risk of death from ischemic heart disease

Supplementary Table S11. Annual death rates per 100,000 during 2010 for women in the 15 Western European countries by age.*

Disease category (ICD-10 code)	**35-39**	**40-44**	**45-49**	**50-54**	**55-59**	**60-64**	**65-69**	**70-74**	**75-79**	**80+/unk.**
Ischaemic heart disease (I20-I25)	1.3	3.3	6.1	11.8	19.1	34.2	66.9	131.7	288.7	1202.3
Heart failure (I50)	0.3	0.4	0.8	1.5	2.5	4.9	10.2	25.9	74.5	561.4
Valvular heart disease (I00-I09, I34-I39, excl I00.9, I01.0, & I09.2)	0.2	0.4	0.6	1.2	2.1	4.6	9.1	21.3	49.4	172.3
Other cardiac diseases	1.5	2.3	3.5	5.6	9.0	15.0	27.4	57.1	143.2	814.3
ALL HEART DISEASES (I00-I09, I11, I13, I20-I51, excl I26)	3.3	6.4	11.0	20.1	32.7	58.7	113.6	236.0	555.8	2750.3
Cerebrovascular disease (I60-I69)	2.0	3.5	6.7	10.4	14.1	21.6	44.5	99.3	247.2	1094.7
Thromboembolic disease (I26, I74, I80-I83)	0.9	1.4	2.4	2.7	3.7	5.7	10.4	17.7	34.0	108.9
Peripheral vascular disease (I70-I73, I77-I79, I83-I87)	0.3	0.6	0.8	1.5	2.7	4.8	9.1	18.7	40.1	182.6
Other vascular diseases	0.1	0.3	0.5	0.7	1.3	1.9	4.0	8.1	19.8	119.0
ALL VASCULAR DISEASES (I00-I99)	6.6	12.2	21.4	35.4	54.5	92.7	181.6	379.8	896.9	4255.5
ALL CAUSES OF DEATH	57.3	95.3	157.6	256.4	375.8	549.7	869.1	1389.8	2623.1	9386.6
**Population (1000s; UN, 2010 revision)**	**13982**	**15276**	**15135**	**13633**	**12495**	**12041**	**10015**	**9810**	**8240**	**13109**

*Austria, Belgium, Denmark, Finland, France, Germany, Greece, Ireland, Italy, Luxembourg, Netherlands, Portugal, Spain, Sweden, United Kingdom

Death counts obtained from WHO, http://www.who.int/healthinfo/morttables/en/, and population counts from UN, http://esa.un.org/unpd/wpp/Excel-Data/population.htm, (both accessed 3 December 2012). For Belgium and Denmark 2006 death rates applied to 2010 population estimates, likewise for France, Greece, Italy and Luxembourg but using 2009 death rates. For Greece, rates for heart failure, valvular, thromboembolic and peripheral vascular disease obtained by subdividing the rates for the remaining vascular diseases in proportion to the age-specific rates in 2010 for women in the other 14 countries.

Supplementary Table 2 Data from population-based survey: mean heart dose and cumulative risk of death from ischemic heart disease

Supplementary Table S12. Cumulative risk of death from ischaemic heart disease (IHD) and absolute risk of radiation-related IHD death by age 80 years according to mean heart dose from breast cancer radiotherapy and age at irradiation, for women with no cardiac risk factors at time of irradiation and for women with at least one cardiac risk factor. Death rates from IHD and from all causes assumed to be equal to recent values for the 15 countries of Western Europe (see Supplementary Table S11). The distribution of cardiac risk factors is assumed to be equal to that in the present study.

		No cardiac risk factor					At least one cardiac risk factor				
		Cumulative risk (%) by attained age				Absolute risk (%) of radiation-related IHD death by age 80 years	Cumulative risk (%) by attained age				Absolute risk (%) of radiation-related IHD death by age 80 years
Age at irradiation (years)	Mean heart dose (Gy)	50	60	70	80		50	60	70	80	
40	0	0.03	0.1	0.5	2.0	0.0	0.08	0.3	0.9	3.3	0.0
	0.5	0.03	0.2	0.5	2.0	0.0	0.09	0.3	0.9	3.4	0.1
	1	0.03	0.2	0.6	2.1	0.1	0.09	0.3	1.0	3.5	0.2
	2	0.03	0.2	0.6	2.2	0.2	0.10	0.3	1.0	3.8	0.5
	3	0.03	0.2	0.6	2.4	0.4	0.10	0.3	1.1	4.0	0.7
	4	0.03	0.2	0.7	2.5	0.5	0.11	0.4	1.2	4.2	0.9
	5	0.04	0.2	0.7	2.7	0.7	0.11	0.4	1.2	4.5	1.2
	6	0.04	0.2	0.8	2.8	0.8	0.12	0.4	1.3	4.7	1.4
	7	0.04	0.2	0.8	3.0	1.0	0.13	0.4	1.4	4.9	1.6
	8	0.04	0.2	0.8	3.1	1.1	0.13	0.5	1.4	5.2	1.9
	9	0.04	0.2	0.9	3.2	1.2	0.14	0.5	1.5	5.4	2.1
	10	0.05	0.3	0.9	3.4	1.4	0.15	0.5	1.6	5.6	2.3
50	0		0.09	0.5	1.9	0.0		0.3	0.9	3.4	0.0
	0.5		0.09	0.5	2.0	0.1		0.3	1.0	3.5	0.1
	1		0.09	0.5	2.1	0.2		0.3	1.0	3.6	0.2
	2		0.10	0.5	2.2	0.3		0.3	1.1	3.8	0.4
	3		0.11	0.6	2.4	0.5		0.3	1.1	4.1	0.7
	4		0.11	0.6	2.5	0.6		0.4	1.2	4.3	0.9
	5		0.12	0.6	2.7	0.8		0.4	1.3	4.6	1.2
	6		0.12	0.7	2.8	0.9		0.4	1.3	4.8	1.4
	7		0.13	0.7	2.9	1.0		0.4	1.4	5.0	1.6
	8		0.14	0.7	3.1	1.2		0.4	1.5	5.3	1.9
	9		0.14	0.8	3.2	1.3		0.5	1.5	5.5	2.1
	10		0.15	0.8	3.4	1.5		0.5	1.6	5.8	2.4
60	0			0.2	1.6	0.0			0.7	3.0	0.0
	0.5			0.2	1.7	0.1			0.7	3.1	0.1
	1			0.2	1.7	0.1			0.8	3.2	0.2
	2			0.3	1.8	0.2			0.8	3.4	0.4
	3			0.3	2.0	0.4			0.9	3.7	0.7
	4			0.3	2.1	0.5			0.9	3.9	0.9
	5			0.3	2.2	0.6			1.0	4.1	1.1
	6			0.3	2.3	0.7			1.0	4.3	1.3
	7			0.3	2.4	0.8			1.1	4.5	1.5
	8			0.4	2.6	1.0			1.1	4.7	1.7
	9			0.4	2.7	1.1			1.2	5.0	2.0
	10			0.4	2.8	1.2			1.3	5.2	2.2
70	0				0.9	0.0				2.7	0.0
	0.5				0.9	0.0				2.8	0.1
	1				0.9	0.0				2.9	0.2
	2				1.0	0.1				3.1	0.4
	3				1.0	0.1				3.3	0.6
	4				1.1	0.2				3.5	0.8
	5				1.2	0.3				3.7	1.0
	6				1.2	0.3				3.9	1.2
	7				1.3	0.4				4.1	1.4
	8				1.4	0.5				4.3	1.6
	9				1.4	0.5				4.5	1.8
	10				1.5	0.6				4.7	2.0

Supplementary Table 3 Data from population-based survey: mean heart dose and cumulative risk of ischemic heart disease

Supplementary Table S13. Cumulative risk of at least one acute coronary event (ACE, ie, a non-fatal or fatal MCE or unstable angina) and absolute risk of at least one radiation-related ACE by age 80 years according to mean heart dose from breast cancer radiotherapy and age at irradiation, for women with no cardiac risk factors at time of irradiation and for women with at least one cardiac risk factor. ACE rate assumed to be 6 times, 5 times, 3 times, and twice the IHD death rate at ages <50, 50-59, 60-69, and 70+ respectively. The distribution of cardiac risk factors is assumed to be equal to that in the present study.

		No cardiac risk factor					At least one cardiac risk factor				
		Cumulative risk (%) by attained age				Absolute risk (%) of radiation-related disease by age 80 years	Cumulative risk (%) by attained age				Absolute risk (%) of radiation-related disease by age 80 years
Age at irradiation (years)	Mean heart dose (Gy)	50	60	70	80		50	60	70	80	
40	0	0.2	0.8	1.9	4.7	0.0	0.5	1.5	3.3	7.9	0.0
	0.5	0.2	0.8	1.9	4.9	0.2	0.5	1.6	3.5	8.2	0.3
	1	0.2	0.8	2.0	5.0	0.3	0.5	1.6	3.6	8.5	0.6
	2	0.2	0.9	2.1	5.4	0.7	0.6	1.7	3.8	9.0	1.1
	3	0.2	0.9	2.3	5.7	1.0	0.6	1.8	4.1	9.6	1.7
	4	0.2	1.0	2.4	6.0	1.3	0.6	1.9	4.3	10.1	2.2
	5	0.2	1.0	2.6	6.4	1.7	0.7	2.0	4.5	10.7	2.8
	6	0.2	1.1	2.7	6.7	2.0	0.7	2.2	4.8	11.2	3.3
	7	0.2	1.1	2.8	7.0	2.3	0.8	2.3	5.0	11.8	3.9
	8	0.2	1.2	3.0	7.4	2.7	0.8	2.4	5.3	12.3	4.4
	9	0.3	1.3	3.1	7.7	3.0	0.8	2.5	5.5	12.8	4.9
	10	0.3	1.3	3.2	8.0	3.3	0.9	2.6	5.7	13.4	5.5
50	0		0.4	1.6	4.5	0.0		1.4	3.3	8.0	0.0
	0.5		0.4	1.6	4.6	0.1		1.4	3.4	8.3	0.3
	1		0.5	1.7	4.8	0.3		1.5	3.5	8.5	0.5
	2		0.5	1.8	5.1	0.6		1.6	3.7	9.1	1.1
	3		0.5	1.9	5.4	0.9		1.7	4.0	9.7	1.7
	4		0.6	2.0	5.8	1.3		1.8	4.2	10.2	2.2
	5		0.6	2.2	6.1	1.6		1.9	4.5	10.8	2.8
	6		0.6	2.3	6.4	1.9		2.0	4.7	11.3	3.3
	7		0.7	2.4	6.7	2.2		2.1	4.9	11.8	3.8
	8		0.7	2.5	7.0	2.5		2.2	5.2	12.4	4.4
	9		0.7	2.6	7.3	2.8		2.3	5.4	12.9	4.9
	10		0.7	2.7	7.7	3.2		2.4	5.6	13.5	5.5
60	0			0.7	3.4	0.0			2.1	6.6	0.0
	0.5			0.7	3.5	0.1			2.2	6.8	0.2
	1			0.7	3.7	0.3			2.3	7.1	0.5
	2			0.8	3.9	0.5			2.5	7.6	1.0
	3			0.8	4.2	0.8			2.6	8.0	1.4
	4			0.9	4.4	1.0			2.8	8.5	1.9
	5			0.9	4.6	1.2			2.9	8.9	2.3
	6			1.0	4.9	1.5			3.1	9.4	2.8
	7			1.0	5.1	1.7			3.2	9.9	3.3
	8			1.1	5.4	2.0			3.4	10.3	3.7
	9			1.1	5.6	2.2			3.6	10.8	4.2
	10			1.2	5.9	2.5			3.7	11.2	4.6
70	0				1.7	0.0				5.4	0.0
	0.5				1.8	0.1				5.6	0.2
	1				1.8	0.1				5.8	0.4
	2				2.0	0.3				6.2	0.8
	3				2.1	0.4				6.6	1.2
	4				2.2	0.5				6.9	1.5
	5				2.3	0.6				7.3	1.9
	6				2.5	0.8				7.7	2.3
	7				2.6	0.9				8.1	2.7
	8				2.7	1.0				8.4	3.0
	9				2.8	1.1				8.8	3.4
	10				3.0	1.3				9.2	3.8

Supplementary Table 4 Lifetime risk of ischemic heart disease in female general population in the Framingham Heart Study, by major cardiac risk factor

Risk stratum	Cumulative IHD risk to 80-year-old, %			Reference
	40-year-old	50-year-old	60-year-old	
**Overall (general-population)[a]**	22.0	20.5	17.0	Lloyd-Jones et al. (23)
**Major cardiac risk factor[b]**				
**Total cholesterol ≥ 240 mg/dL**	-	34.0	28.3	Lloyd-Jones et al. (24)
**Hypertension (grade II-III)**	-	42.0	38.9	Lloyd-Jones et al. (24)
**Smoker**	-	25.0	21.8	Lloyd-Jones et al. (24)
**Diabetes**	-	> 57.3	> 46.6	Lloyd-Jones et al. (24)
**≥ 2 major cardiac risk factors**	-	40.2	30.5	Lloyd-Jones et al. (24)

IHD, ischemic heart disease.

[a]The IHD risk of overall level in the Framingham Heart Study.

[b]Major cardiac risk factor is defined as total cholesterol ≥ 240 mg/dL, grade II-III hypertension (systolic blood pressure ≥ 160 mm Hg or diastolic blood pressure ≥ 100 mm Hg), smoker, or diabetic (24).

Supplementary Table 5 Cost-effectiveness and photon mean heart dose in 40-year-old patients without preexisting cardiac risk factor

Photon MHD, Gy	Relative risk[a]	Cumulative IHD risk to 80-year-old[b], %				ICER, $/QALY			
		Proton (MHD: 0.5Gy)		Photon		Proton cost			
		IHD death	IHD[c]	IHD death	IHD	$50,000	$40,000	$30,000	$20,000
1	0.784	2.0	4.9	2.1	5.0	5,980,823.4	4,406,977.5	2,833,131.6	1,259,285.7
2	0.838	2.0	4.9	2.2	5.4	2,779,338.2	2,047,318.9	1,315,299.6	583,280.2
3	0.892	2.0	4.9	2.4	5.7	1,688,861.3	1,243,893.1	798,925.0	353,956.9
4	0.946	2.0	4.9	2.5	6.0	1,310,522.7	965,032.4	619,542.0	274,051.7
5	1	2.0	4.9	2.7	6.4	975,140.6	717,908.5	460,676.4	203,444.3
6	1.054	2.0	4.9	2.8	6.7	837,311.2	616,305.5	395,299.7	174,294.0
7	1.108	2.0	4.9	3.0	7.0	701,802.4	516,509.1	331,215.9	145,922.6
8	1.162	2.0	4.9	3.1	7.4	613,271.0	451,202.6	289,134.2	127,065.9
9	1.216	2.0	4.9	3.2	7.7	554,912.9	408,164.6	261,416.4	114,668.2
10	1.27	2.0	4.9	3.4	8.0	489,870.7	360,292.9	230,715.0	101,137.1
11	1.324	2.0	4.9	3.4	8.5	451,409.9	331,801.1	212,192.2	92,583.4
12	1.378	2.0	4.9	3.6	8.9	401,709.3	295,207.2	188,705.1	82,203.0
13	1.432	2.0	4.9	3.7	9.2	375,303.8	275,741.3	176,178.7	76,616.2
14	1.486	2.0	4.9	3.9	9.6	340,216.3	249,901.2	159,586.0	69,270.9
15	1.54	2.0	4.9	4.0	9.9	320,175.1	235,123.3	150,071.5	65,019.7
16	1.594	2.0	4.9	4.1	10.3	299,846.3	220,120.1	140,393.9	60,667.8

MHD, mean heart dose; Gy, Gray; IHD, ischemic heart disease; ICER, incremental cost-effectiveness ratio; $, US dollars; QALY, quality-adjusted life-year.

[a]The relative risk in comparison to the baseline IHD risk (40-year-old, a photon MHD of 5Gy, without preexisting cardiac risk factor).

[b]Model calibrations results for IHD risk in Markov models, which were set to be exactly the same as the data of Darby et al. (15); the risk of IHD increased linearly with MHD by 7.4% per Gy, compared with the MHD of 0 Gy.

[c]The IHD included IHD death, nonfatal acute myocardial infarction, unstable angina pectoris, and ischemic heart failure.

Supplementary Table 6 Cost-effectiveness and preexisting cardiac risk in 40-year-old patients with a photon mean heart dose of 5 Gy

Relative risk[a]	Cumulative IHD risk to 80-year-old[b], %				ICER, $/QALY			
	Proton (MHD: 0.5Gy)		Photon (MHD: 5Gy)		Proton cost			
	IHD death	IHD[c]	IHD death	IHD	$50,000	$40,000	$30,000	$20,000
1	2.0	4.9	2.7	6.4	975,140.6	717,908.5	460,676.4	203,444.3
2	4	9.8	5.4	12.8	476,919.6	350,782.9	224,646.1	98,509.4
3	6	14.7	8.1	19.2	300,835.9	221,024.3	141,212.7	61,401.1
4	8	19.6	10.8	25.6	209,825.6	153,950.0	98,074.3	42,198.6
5	10	24.5	13.5	32	155,515.7	113,916.3	72,317.0	30,717.6
6	12	29.4	16.2	38.4	114,416.9	83,594.5	52,772.1	21,949.7
7	14	34.3	18.9	44.8	86,942.0	63,349.5	39,757.1	16,164.6
8	16	39.2	21.6	51.2	63,309.9	45,918.1	28,526.3	11,134.5
9	18	44.1	24.3	57.6	42,073.9	30,239.2	18,404.4	6,569.7
10	20	49	27	64	27,681.8	19,559.1	11,436.4	3,313.7

MHD, mean heart dose; Gy, Gray; IHD, ischemic heart disease; ICER, incremental cost-effectiveness ratio; $, US dollars; QALY, quality-adjusted life-year.

[a]The relative risk in comparison to the baseline IHD risk (40-year-old, a photon MHD of 5Gy, without preexisting cardiac risk factor).

[b]Model calibrations results for IHD risk in Markov models, which were set to be exactly the same as the data of Darby et al. (15).

[c]The IHD included IHD death, nonfatal acute myocardial infarction, unstable angina pectoris, and ischemic heart failure.

Supplementary Table 7 Cost-effectiveness and photon mean heart dose in 50-year-old patients without preexisting cardiac risk factor

Photon MHD (Gy)	Relative risk[a]	Cumulative IHD risk to 80-year-old[b], %				ICER, $/QALY			
		Proton (MHD: 0.5Gy)		Photon		Proton cost			
		IHD death	IHD[c]	IHD death	IHD	$50,000	$40,000	$30,000	$20,000
1	0.784	2.0	4.6	2.1	4.8	4,715,934.9	3,474,863.0	2,233,791.0	992,719.1
2	0.838	2.0	4.6	2.2	5.1	2,748,470.4	2,024,905.3	1301,340.3	577,775.3
3	0.892	2.0	4.6	2.4	5.4	1,528,810.0	1,126,054.9	723,299.9	320,544.8.
4	0.946	2.0	4.6	2.5	5.8	1,203,037.7	885,971.2	568,904.6	251,838.0
5	1	2.0	4.6	2.7	6.1	879,729.9	647,703.4	415,676.9	183,650.5
6	1.054	2.0	4.6	2.8	6.4	770,855.9	567,466.6	364,077.4	160,688.1
7	1.108	2.0	4.6	2.9	6.7	686,038.1	504,958.5	323,878.9	142,799.3
8	1.162	2.0	4.6	3.1	7.0	562,450.6	413,878.2	265,305.8	116,733.3
9	1.216	2.0	4.6	3.2	7.3	516,039.2	379,674.3	243,309.4	106,944.5
10	1.27	2.0	4.6	3.4	7.7	443,033.8	325,871.3	208,708.8	91,546.4
11	1.324	2.0	4.6	3.4	8.2	413,806.6	304,331.7	194,856.7	85,381.7
12	1.378	2.0	4.6	3.6	8.5	388,221.1	285,475.8	182,730.5	79,985.2
13	1.432	2.0	4.6	3.7	8.8	365,636.6	268,831.6	172,026.5	75,221.5
14	1.486	2.0	4.6	3.9	9.2	327,580.2	240,784.9	153,989.5	67,194.2
15	1.54	2.0	4.6	4.0	9.5	309,869.1	227,732.2	145,595.2	63,458.3
16	1.594	2.0	4.6	4.1	9.8	280,919.6	206,396.9	131,874.3	57,351.6

MHD, mean heart dose; Gy, Gray; IHD, ischemic heart disease; ICER, incremental cost-effectiveness ratio; $, US dollars; QALY, quality-adjusted life-year.

[a]The relative risk in comparison to the baseline IHD risk (50-year-old, a photon MHD of 5Gy, without preexisting cardiac risk factor).

[b]Model calibrations results for IHD risk in Markov models, which were set to be exactly the same as the data of Darby et al. (15); the risk of IHD increased linearly with MHD by 7.4% per Gy, compared with the MHD of 0 Gy.

[c]The IHD included IHD death, nonfatal acute myocardial infarction, unstable angina pectoris, and ischemic heart failure.

Supplementary Table 8 Cost-effectiveness and preexisting cardiac risk in 50-year-old patients with a photon mean heart dose of 5 Gy

Relative risk[a]	Cumulative IHD risk to 80-year-old[b], %				ICER, $/QALY			
	Proton (MHD: 0.5Gy)		Photon (MHD: 5Gy)		Proton cost			
	IHD death	IHD[c]	IHD death	IHD	$50,000	$40,000	$30,000	$20,000
1	1.9	4.5	2.7	6.1	879,729.9	647,703.4	415,676.9	183,650.5
2	3.8	9.0	5.4	12.2	402,038.1	295,702.9	189,369.6	83,032.4
3	5.7	13.5	8.1	18.3	255,721.6	187,879.9	120,038.2	52,196.5
4	7.6	18	10.8	24.4	183,154.8	134,417.2	85,679.5	36,941.8
5	9.5	22.5	13.5	30.5	137,207.9	100,563.0	63,918.1	27,273.3
6	11.4	27	16.2	36.6	105,916.2	77,507.4	49,098.5	20,689.7
7	13.3	31.5	18.9	42.7	84,174.9	61,464.2	38,753.5	16,042.7
8	15.2	36	21.6	48.8	67,923.6	49,437.4	30,951.2	12,465.0
9	17.1	40.5	24.3	54.9	52,073.5	37,785.7	23,497.9	9,210.1
10	19	45	27	61	39,299.2	28,356.2	17,413.1	6,470.0

MHD, mean heart dose; Gy, Gray; IHD, ischemic heart disease; ICER, incremental cost-effectiveness ratio; $, US dollars; QALY, quality-adjusted life-year.

[a]The relative risk in comparison to the baseline IHD risk (50-year-old, a photon MHD of 5Gy, without preexisting cardiac risk factor).

[b]Model calibrations results for IHD risk in Markov models, which were set to be exactly the same as the data of Darby et al. (15).

[c]The IHD included IHD death, nonfatal acute myocardial infarction, unstable angina pectoris, and ischemic heart failure.

Supplementary Table 9 Cost-effectiveness and photon mean heart dose in 60-year-old patients without preexisting cardiac risk factor

Photon MHD (Gy)	Relative risk[a]	Cumulative IHD risk to 80-year-old[b], %				ICER, $/QALY			
		Proton (MHD: 0.5Gy)		Photon		Proton cost			
		IHD death	IHD[c]	IHD death	IHD	$50,000	$40,000	$30,000	$20,000
1	0.784	1.7	3.5	1.7	3.7	9,711,490.9	7,154,698.6	4,597,906.2	2041,113.8
2	0.838	1.7	3.5	1.8	3.9	3,992,375.9	2,940,978.5	1,889,581.0	838,183.6
3	0.892	1.7	3.5	2.0	4.2	1,920,303.9	1,414,396.2	908,488.6	402,580.9
4	0.946	1.7	3.5	2.1	4.4	1,525,280.2	1,123,338.5	721,396.8	319,455.2
5	1	1.7	3.5	2.2	4.6	1,258,909.7	927,126.4	595,343.0	263,559.7
6	1.054	1.7	3.5	2.3	4.9	1,014,418.3	746,883.7	479,349.1	211,814.6
7	1.108	1.7	3.5	2.4	5.1	876,204.8	645,051.0	413,897.2	182,743.4
8	1.162	1.7	3.5	2.6	5.4	721,862.2	531,334.7	340,807.2	150,279.6
9	1.216	1.7	3.5	2.7	5.6	651,483.6	479,498.6	307,513.7	135,528.7
10	1.27	1.7	3.5	2.8	5.9	583,856.4	429,671.3	275,486.1	121,300.9
11	1.324	1.7	3.5	2.9	6.2	526,935.5	387,701.3	248,467.0	109,232.7
12	1.378	1.7	3.5	3.0	6.4	482,256.8	354,783.6	227,310.5	99,837.4
13	1.432	1.7	3.5	3.1	6.7	446,875.0	328,701.2	210,527.3	92,353.5
14	1.486	1.7	3.5	3.3	6.9	408,941.9	300,781.7	192,621.4	84,461.2
15	1.54	1.7	3.5	3.4	7.2	376,736.2	277,046.7	177,357.1	77,667.5
16	1.594	1.7	3.5	3.5	7.4	356,156.0	261,883.8	167,611.5	73,339.2

MHD, mean heart dose; Gy, Gray; IHD, ischemic heart disease; ICER, incremental cost-effectiveness ratio; $, US dollars; QALY, quality-adjusted life-year.

[a]The relative risk in comparison to the baseline IHD risk (60-year-old, a photon MHD of 5Gy, without preexisting cardiac risk factor).

[b]Model calibrations results for IHD risk in Markov models, which were set to be exactly the same as the data of Darby et al. (15); the risk of IHD increased linearly with MHD by 7.4% per Gy, compared with the MHD of 0 Gy.

[c]The IHD included IHD death, nonfatal acute myocardial infarction, unstable angina pectoris, and ischemic heart failure.

Supplementary Table 10 Cost-effectiveness and preexisting cardiac risk in 60-year-old patients with a photon mean heart dose of 5 Gy

Relative risk[a]	Cumulative IHD risk to 80-year-old[b], %				ICER, $/QALY			
	Proton (MHD: 0.5Gy)		Photon (MHD: 5Gy)		Proton cost			
	IHD death	IHD[c]	IHD death	IHD	$50,000	$40,000	$30,000	$20,000
1	1.7	3.5	2.2	4.6	1,258,909.7	927,126.4	595,343.0	263,559.7
2	3.4	7	4.2	9.2	655,174.1	482,080.9	308,987.7	135,894.6
3	5.1	10.5	6.6	13.8	395,118.2	290,592.6	186,067.0	81,541.4
4	6.8	14	8.8	18.4	290,126.8	213,220.5	136,314.3	59,408.0
5	8.5	17.5	11	23	212,787.3	156,337.3	99,887.3	43,437.3
6	10.2	21	13.2	27.6	182,930.9	134,224.9	85,518.9	36,812.9
7	11.9	24.5	15.4	32.2	148,714.9	108,992.3	69,269.7	29,547.0
8	13.6	28	17.6	36.8	123,775.4	90,600.9	57,426.2	24,251.6
9	15.3	31.5	19.8	41.4	104,011.3	76,030.9	48,050.4	20,070.0
10	17	35	22	46	87,364.7	63,751.9	40,139.2	16,526.4

MHD, mean heart dose; Gy, Gray; IHD, ischemic heart disease; ICER, incremental cost-effectiveness ratio; $, US dollars; QALY, quality-adjusted life-year.

[a]The relative risk in comparison to the baseline IHD risk (60-year-old, a photon MHD of 5Gy, without preexisting cardiac risk factor).

[b]Model calibrations results for IHD risk in Markov models, which were set to be exactly the same as the data of Darby et al. (15).

[c]The IHD included IHD death, nonfatal acute myocardial infarction, unstable angina pectoris, and ischemic heart failure.
